# Complexity of Primary Lifetime Occupation and Cognitive Processing

**DOI:** 10.3389/fpsyg.2019.01861

**Published:** 2019-08-21

**Authors:** Daniel Eriksson Sörman, Patrik Hansson, Ilona Pritschke, Jessica Körning Ljungberg

**Affiliations:** ^1^Department of Psychology, Umeå University, Umeå, Sweden; ^2^Division of Human Work Science, Luleå University of Technology, Luleå, Sweden

**Keywords:** occupational complexity, work complexity, DOT, executive functioning, inhibition, switching, updating, cognition

## Abstract

Today, there are a lack of studies focusing on the relationship between occupational complexity and executive functioning. This is noteworthy since executive functions are core aspects of cognitive processing. The present study was aimed to investigate if three occupational complexity factors (with data, people, and things) of main lifetime occupation were related to performance in executive tasks (inhibition, switching, updating). We analyzed cross-sectional data that were available for 225 participants aged 50–75 years. Results from structural equation models showed that higher complexity levels of working with data were related to lower error rates in the updating component of cognitive control. In addition, higher rates of complexity working with people was associated with lower error rates in task-switching, which also persisted after adjustment of fluid intelligence. Complexity with things, however, was not related to performance in the executive tasks. Future studies would benefit from a longitudinal design to investigate if the results from this study also hold in the long term and to further investigate the directionality between factors.

## Introduction

Today, we know of several factors that can promote cognitive functioning. It has, for instance, been found that engagement in physical and mentally stimulating activities (for reviews see e.g., Fratiglioni et al., 2004; Stern and Munn, 2010; Fallahpour et al., 2016), as well as speaking two or more languages (for reviews, see e.g., Bialystok et al., 2009, 2012), can generate transfer effects and enhance cognitive performance in the lab. It should be noted though that beneficial effects do not seem to be found in all cognitive domains for these factors (see e.g., Boot et al., 2008; Lehtonen et al., 2018). To this date, it has also been found that individuals that are highly educated and have occupations with high mental requirements show better performance in the lab, which have been demonstrated on several global measures of cognitive functioning (e.g., Mini-Mental State Examination) in cross-sectional designs, but also with regard to rate of cognitive change and time of onset for cognitive impairment (Fisher et al., 2014; Vemuri et al., 2014; Pool et al., 2016).

Beneficial effects of occupation on cognitive functioning may be of special interest considering the amount of time many individuals spend on their work, and several job properties have been suggested to preserve or to improve cognitive abilities. Work environments that provide more cognitive stimulation have been found to be positively related to immediate and delayed memory as well as processing speed (Ansiau et al., 2005), and individuals with less prestigious occupations have demonstrated lower scores in several cognitive tasks, including measures of immediate memory, delayed memory, attention, and orientation (Scherr Paul et al., 1988). Flexible work compared to repetitive and routine work have also been found to result in a higher intellectual flexibility and less cognitive decline (Miller et al., 1979; Gajewski et al., 2010). Moreover, forestry, fishing, and craft workers have shown an elevated risk for cognitive impairment compared to former legislators, business executives, and managers, as indicated by lower scores in the Short Portable Mental Status Questionnaires (Li et al., 2002). Other occupational groups have also been investigated. In a study by Van der Elst et al. (2012), the authors found that primary and secondary teachers have better working memory and verbal fluency abilities than participants in other occupations, even when matched for age, gender, occupational, and educational level. Karp et al. (2004) revealed that low education and blue-collar occupations, compared to white-collar occupations, increases the risk of Alzheimer’s disease and dementia.

However, even if it is possible that occupational complexity may preserve or even enhance cognitive abilities, an alternative explanation is that individuals with initially higher cognitive capacity will end up in more complex work situations. Indeed, several studies suggest reciprocal effects (Schooler et al., 1999), demonstrating associations between intellectual flexibility and work complexity (Kohn and Schooler, 1978; Naoi and Schooler, 1985), as well as between intellectual functioning and occupational self-direction (Naoi and Schooler, 1985; Schooler et al., 2004). General mental ability (GMA), a human characteristic assumed to reflect a general measure of cognitive functioning, which works across several different domains, has frequently been studied as predictive of educational level and occupational characteristics (see, e.g., Murray, 1998). GMA has been found to predict work performance in many different occupational areas (for meta analyses, see, e.g., Schmidt and Hunter, 1998; Salgado et al., 2003; Bertua et al., 2005), and is proposed to have even larger validity estimates on work performance than personality traits, such as Conscientiousness and Emotional Stability (Schmidt et al., 2008), which have previously been related to job performance (see e.g., Barrick and Mount, 1991; Hurtz and Donovan, 2000). As a natural consequence of higher educational level and better work performance, GMA has been connected to higher occupational level (Schmidt and Hunter, 2004) and higher income (Murray, 1998). General cognitive ability has been associated to occupational complexity as well, suggesting that the more complex an occupation is, the stronger the relationship is. This association also informs that individuals that have occupations with lower complexity than their cognitive ability would predict, are more likely to move to an occupation that better matches their ability, whereas individuals with low mental ability are more likely to move to an occupation with lower complexity demands (Wilk and Sackett, 1996).

So-called occupational complexity is a work characteristic that has received more interest in relation to cognitive functioning during the last decades (see e.g., Finkel et al., 2009; Andel et al., 2014; Smart et al., 2014; Feldberg et al., 2016). One method commonly used to measure occupational complexity is to use of information included in Dictionary of Occupational Titles (DOT; U.S. Department of Labor, 1977; see also https://occupationalinfo.org/appendxb_1.html). This is a source of occupational information in the United States in which occupations have been defined by job analysts with regard to three complexity dimensions: (1) complexity with people, (2) complexity with data, and (3) complexity with things. Complexity with data, for example, is characterized by operations of analyzing and computing. Occupations with higher complexity of working with people can be associated with more monitoring and supervising, and complexity with things with more handling and precision working (Smart et al., 2014).

By using information about participants’ primary lifetime occupation, Andel et al. (2007) found that higher level of occupational complexity working with people and data, independently, were related to higher scores in the mini-mental state examination in older adulthood. Smart et al. (2014) later confirmed that occupational complexity with people and data was related to better cognitive performance in old age. More precisely, after adjustment for covariates age, sex, age 11 IQ, years of education and deprivation, both complexity with people and data were related to higher general cognitive ability (g) scores. Complexity with data was also related to better performance processing speed, whereas complexity with people was associated with higher memory scores, as well. In a cross-sectional study on participants aged 65 and older, Correa Ribeiro et al. (2013) similarly found evidence of associations between complexity working with data and things and better cognitive performance (MMSE). However, the authors did not find any relationships for occupations related to higher complexity with people. It should be noted that even in patients with Mild Cognitive Impairment (MCI), a more complex main lifetime occupation has been related to better cognitive performance. Feldberg et al. (2016) found that working with data was related to higher performance in attentional tests, and that complexity of working with people could be related to superior verbal ability. Based on three main lifetime occupations, Boots et al. (2015) found among participants at risk of Alzheimer’s disease (AD) that complex work with people was associated to increased brain atrophy and decreased hippocampal volume when participants were matched for cognitive function. Thus, it seem that individuals with a history of complex work with people are more able to cope with AD pathology since they have equal cognitive ability but worse AD pathology compared to those with an occupational history that has lower complexity of working with people.

Many previous findings may suggest that effects of occupational complexity on cognitive functioning are in support of the cognitive reserve hypothesis (Stern, 2002), which posits that environmental enrichment may provide resources to better cope with dementia pathology. The cognitive reserve hypothesis is considered as an active model, suggesting that it is influenced by mental enrichment. However, passive models should also be stressed in this context. These are often linked to the brain reserve concept (Katzman et al., 1988; Katzman, 1993), which rather considers the reserve capacity as the hardware of the brain. The brain reserve concept highlights biological differences as one plausible cause of reserve capacity. It should be noted, however, that the “cognitive reserve hypothesis” and the “brain reserve hypothesis” are not mutually exclusive. Thus, even if individuals with larger brain reserve are better suited to cope with pathology associated with cognitive impairment, it is still possible that cognitive stimulation may cause physiological changes that modify the ability to cope with age-related changes in the brain (Valenzuela, 2008).

Even if results differ between studies, with some not in support of any long-term effects of work complexity on cognition (see e.g., Gow et al., 2014), overall most studies have revealed both short-term and long-term relationships, at least from one or two complexity measures. Thus, it is likely to consider that there are at least some long-term associations between professions with a higher level of occupational complexity on cognitive functioning. So far, however, only a limited number of studies have investigated occupational complexity in relation to executive control. This is noteworthy since attentional resources are much needed in everyday life, but also as performance in tasks used to measure executive functioning and its subcomponents starts to decline at a relatively early age (see e.g., Salthouse and Miles, 2002; Zelazo et al., 2004; Treitz et al., 2007). The subcomponents of executive functions often referred to in the literature are inhibition, switching, and updating. Inhibition is assumed to reflect the ability to supress prepotent conflicting responses in a given situation (Morris and Jones, 1990; Miyake et al., 2000), switching is understood as the ability to switch between different tasks or mental states (Miyake et al., 2000; Monsell, 2003), and updating refers to being able to quickly monitor and evaluate incoming information for task-relevance and use this new information to revise the information used in one’s working memory, if needed (Morris and Jones, 1990; Miyake et al., 2000). All of these three sub-processes are likely to be activated to different degrees during certain types of job demands, but to our knowledge only verbal fluency so far has been applied as an indicator of executive functioning (Adam et al., 2013). Although fluency tasks can be reflective also of other processes such as semantic memory and processing speed, the results were interesting, showing that professional activity (employed or self-employed), compared to periods of work inactivity, was related to better performance. Similar associations were, as previously noted, obtained when comparing teachers and non-teachers on verbal fluency and working memory (Van der Elst et al., 2012).

It seems plausible that several work characteristics, such as consulting or teaching, put high demands on the executive control system. A high school teacher, for instance, must *switch* between teaching on a course, answering questions of pupils, and performing supervision of students. Certainly, such work demands require the ability to be able switch between tasks and mental states. A project manager must be capable of *switching* attention to lead employees, answering customer questions, and keeping track of finances, and also doing this in several projects at the same time. However, both these examples not only illustrate switches between tasks. Each task (e.g., teaching, supervising, answering questions, keeping track of finances) in itself also requires the ability to continuously *update* your memory with new information, as well as to be able to focus on what is relevant at the moment and *inhibit* any information irrelevant to solve the task. As occupations have different manifestations of work complexity, one could assume that people with more complex work also would show better performance in executive tasks.

That cognitive stimulation in one’s work environment can cause long-term beneficial effects on the executive control system is not given, though. Results from cognitive training studies have shown that it is difficult to demonstrate far transfer (i.e., beneficial effects also to other tasks than those specifically trained) and long-term maintenance of cognitive training, which must be seen as one of the core aspects to understand the effectiveness of training interventions (Hertzog et al., 2008). For instance, Sandberg (2014) found in a study of young (*M* = 27.5) and old (*M* = 71.6) participants, who performed executive process training, that transfer effects that persisted over a period of 18 months were present only in tasks with a substantial process overlap to those tasks trained. Thus, so-called far transfer effects were not found over time for any of the age groups. Therefore, more knowledge is needed about possible gains of cognitive stimulation on the executive control system. One way to move forward may be to study long-term, ecologically-valid cognitive training, such as the impact of mentally demanding occupations across the life course.

Given the limited number of studies that have investigated the relationship between occupational complexity and executive functioning, and the lack of studies using a wider set of executive tests to measure the sub-components of executive functions, this area of research needs to be broadened. The aim of this study was to explore if any of the complexity dimensions included in DOT (complexity with people, data, or things) of main lifetime occupation were related to performance in executive functioning (switching, inhibition, updating) in a sample of 50–75 year-old participants. Structural equation modeling was used, and since it is well-documented that education and occupation are closely-related factors (see e.g., White et al., 1994), but can still have independent effects on cognitive functioning, both of these factors were considered in the analyses. In addition, both age and gender were included in the analyses, since these factors have been related to performance in the domain of cognitive control (see, e.g., Zelazo et al., 2004; Upadhayay and Sanjeev, 2014).

## Materials and Methods

### Participants

The data used in the present study emanates from a project called “Successful aging–A study of how bilingualism and choice of occupation contribute to preserve attention and memory across the adult life span,” which is an ongoing study in Umeå, Sweden. The study has been approved by the Regional Ethics Committee at Umeå University (2016/101-31Ö) and all subjects gave written informed consent in accordance with the Declaration of Helsinki. The participants, all between 50–75 years, were recruited via advertising in local newspapers and through pensioners’ associations. They were invited to participate over two test sessions, about 1 week apart, both with focus on assessment of cognitive functions. In total, 240 neurologically healthy participants contributed with information about their main lifetime occupation. For 225 of them, 136 women and 89 men, it was possible to link main occupation to the DOT codings that were used to categorize occupation complexity in the present study.

### Measures

#### Occupational Complexity

As part of an occupational history questionnaire, participants were asked to provide information about their longest held main occupation. This included information about occupational title, task specifications, and the number of years in this occupation. Main occupation was then matched with the best fitting category listed in the fourth edition of the U.S. Dictionary of Occupational Titles (DOT; U.S. Department of Labor, 1977). In DOT, more than 12,000 occupations have been evaluated based on observations by job analysts. Occupations are classified based on a 9-digit code (e.g., 354.374-010, nurse), and the three digits in the middle represent occupational complexity with data, people, and things, respectively. Scores were coded so that for each dimension a higher value was indicative of higher complexity (ranges for data 0–6; for people 0–8, for things 0–7). The coding and categorization of worker activities into working with data, people, and things has been used (see e.g., Smart et al., 2014; Boots et al., 2015; Feldberg et al., 2016) and validated in previous studies and is therefore a useful tool for classifying work requirements (Kohn and Schooler, 1983; Peterson et al., 2001). Dimensions used in classification of occupations can be seen in Table 1.

**TABLE 1:** Dimensions used in the rating of occupations into complexity of working with data, people, and things.

**Data**		**People**		**Things**	
6	Synthesizing	8	Mentoring	7	Setting Up
5	Coordinating	7	Negotiating	6	Precision Working
4	Analyzing	6	Instructing	5	Operating-Controlling
3	Compiling	5	Supervising	4	Driving-Operating
2	Computing	4	Diverting	3	Manipulating
1	Copying	3	Persuading	2	Tending
0	Comparing	2	Speaking – Signaling	1	Feeding-Offbearing
		1	Serving	0	Handling
		0	Taking Instructions-Helping		

*Reference from the Dictionary of Occupational Titles (DOT). Ratings have been reversed so that a higher value is indicative of greater complexity.*

#### Executive Functioning

The computerized tasks used in the present study were programed in E-Prime 2.0 professional (Schneider et al., 2002). In all tasks, participants were instructed to respond as quickly and accurately as possible.

##### Inhibition

Three tasks were used to measure inhibition. The Flanker task (Eriksen and Eriksen, 1974) was the first task used as an indicator of inhibitory control. A fixation cross (+) was displayed for 2000 ms followed by five arrows shown at the center of the screen (i.e., < < > < <). Participants were instructed to ignore the direction of the flanker arrows and respond to whether the arrow at the center of was pointing to the right (pressed “M” key) or the left (pressed “X” key). In congruent conditions, the central target points in same direction as the flanker arrows. In incongruent conditions, the central target points in the opposite direction as the flanker arrows. The task started with 10 practice trials followed by 96 (6^*^16) test trials. Half of the trials were congruent conditions and half incongruent. If no response was given, the stimuli remained for 2000 ms on the screen. Two measures were used as indicators of performance: (1) The difference in mean response time (RT) between congruent and incongruent trials (the so-called Flanker Effect), and (2) the number of total errors.

The Stroop task (Stroop, 1935; Lu and Proctor, 1995) was the second task used as a measure of inhibition. In this task, a fixation was first displayed for 600 ms followed by a “color” word. That task was to identify the ink of the written color word. In congruent trials, the color of the ink in which the word is written matches the color name (e.g., blue written in blue ink). In incongruent trials, the ink did not match the name of the color (e.g., yellow written in green ink). Participants were given two alternative answers, one on each side of the stimulus, and were instructed to press the “M” key if they thought the alternative on the right was the correct, and the “X” key for the alternative to the left. After a response was made, the next trial started. The task started with six practice trials followed by 96 (2^*^48) test trials. Two performance measures were used: (1) The difference in mean RTs between congruent and incongruent trials (the Stroop effect), and (2) total errors.

The Simon task (Simon and Wolf, 1963) was the third task used to measure inhibitory control. This version of the task was programed based on the one described by Bialystok et al. (2004). A fixation cross was displayed for 800 ms, followed by a 250-ms blank interval, and then a green or red square was presented either on the right or left side of the screen. The task was to identify the color of the square and press the “X” key, located on the left side of the keyboard, if the square was red, and press the “M” key, located on the right side of the keyboard, if it was green. In congruent conditions, the square was presented on the same side of the screen as the associated response key on the keyboard (i.e., red square on the left side of the screen). In incongruent trials, the square was on the opposite side as the associated response key (i.e., red square on the right side of the screen). Participants had to respond within 1000 ms. Each trial was separated by a 500 ms response-to-stimulus interval. The task started with 20 practice trials followed by 80 (2^*^40) test trials. Half of the trials were incongruent trials and half congruent trials. Similar to the other inhibitory tasks used in this study, two measures were used: (1) The difference in mean RTs between congruent and incongruent trials (the Simon effect), and (2) the number of errors.

##### Switching

Three tasks were used to measure switching. The Number-Letter task (Rogers and Monsell, 1995) was the first task used as a measure of switching ability. This was a modified version in which a pair of one number and one letter (e.g., 7A) was presented in one of the four corners on the computer screen. If the pair appeared in any corner at the top of the screen, the participant had to decide if the number was even or odd by pressing the “X” key for odd and the “M” key for even. If the pair appeared in either of the bottom corners of the screen, they had to decide if the letter was in lower or in upper case by pressing the “X” key for a lower case letter and “M” key for an upper case letter. Three test blocks were performed, each of which was preceded by eight practice trials. In the first block (32 trials), stimuli were presented only in the top corners of the screen, and thus, participants categorized only numbers. In the second block (32 trials), stimuli appeared only in the bottom of the screen, and consequently participants made decisions only on the letters. In the third and final mixed block (128 trials), pairs rotated clockwise around the screen and thus a mental shift was required between classification of numbers and letters. Two measures were used: (1) the difference in average RT between switching trials in the third block (mental shift required) and non-switching trials (no shift required) as measure of processing cost, and (2) the number incorrect responses (total errors).

The Color-Shape task, similar to the version used by Prior and Macwhinney (2010), was used as the second measure of switching function. A fixation cross was presented at the center of the screen for 350 ms. Then a blank screen was shown for 150 ms, followed by a figure. In all trials, the figure was either a blue circle, a blue triangle, a red circle, or a red triangle. The task included several blocks. In the first (36 trials), participants were instructed to identify the color of the figure. If it was blue, participants were told to press the “Z” key with their left middle finger. If it was red, they were instructed to press the “X” key with their left index finger. In the second block (36 trials), participants made shape decisions. If the figure was a triangle, participants were instructed to press the “M” key with their right middle finger, and if it was a circle to press the “N” key with their left index finger. Finally, after 16 practice trials, participants performed three mixed-task blocks (3^*^48 trials). In the mixed conditions, a pre-cue was shown for 250 ms, and then the stimulus was presented and remained above the figure until a response was given. Participants made color decisions of the figure if the pre-cue was a rainbow, and shape decisions if the pre-cue was a black circle embedded within a black triangle. Half of the trials were switching conditions and half non-switching conditions. Two measures of switching ability were used: (1) The difference in mean RTs between switching and non-switching trials in the mixed task blocks, and (2) total errors.

The Local-Global task (Navon, 1977), similar to the one used by Miyake et al. (2000), was used as the third measure of switching ability. A fixation cross was shown for 350 ms before a figure appeared on the screen, that was either a cross, a triangle, a square, or a circle. Each “global” figure shown on the screen was in turn built up by smaller “local” figures. The “local” figures could either be consistent or inconsistent with the shape of the “global” figure. If it was blue, participants had to decide shape of the global figure. If it was black, participants decided shape of the local figures. If participants thought that the correct answer was “circle” they pressed the “1” key (i.e., 1 line). For a cross they pressed the “2” key (2 lines), for a triangle the “3” key (3 lines), and for square the “4” key (4 lines). Each trial was separated by a 500 ms response-to-stimulus interval and participants performed 38 practice trials before the test including 98 test trials started. Mental switch was required when switching from categorizing a “local” figure to a “global” figure, and vice versa. Non-switch trials were those when participants continued to do the same categorization as in previous stimuli. The test had an equal amount of switch and non-switch trials. The two switch-cost measures used were (1) the difference in mean RTs between switch trials and non-switch trials and (2) the number of errors.

##### Updating

Three tasks were used to measure updating. The N-back task (Kirchner, 1958) was the first task used as a measure of updating ability. In this task, numbers were displayed on the screen, one number at a time. The task was to determine if the number displayed on the screen was identical (yes/no) to the number presented two steps earlier. If “yes,” participants were instructed to press the “M” key. If “no,” to press the “X” key (e.g., 88 = no, 3 = no, 88 = yes, 52 = no, 3 = no, 52 = yes). Each number was displayed for 2500 ms at the center of the screen followed by a 2000 ms blank interval. After 15 practice trials, the participants performed 40 test trials. The number of errors were used as dependent variable in the analyses.

Matrix monitoring (Salthouse et al., 2003) was the second task used to measure the updating function. Two grids were displayed on the screen, one grid at the top half of the screen, the other on the bottom half of the screen. Each grid had 16 boxes, and in both of them there was one black dot. First the participants memorized the location of the dot in both grips for 2500 ms. Then, both grids disappeared and arrows were displayed for 1200 ms in random order both at the top and the bottom of the screen (arrows were not shown not simultaneously). Each arrow was separated by a 250 ms response-to-stimulus interval. Arrows could point in four different directions; left, up, right, or down. If an arrow was shown at the top of the screen, participants had to visualize how the dot moved one step in upper grid. If an arrow was displayed at bottom of the screen, participants had to visualize movement of one step for the dot in the lower grid. Thus, for both grids participants continuously had to update their memory about the location of the dot. At the end of each trial, participants were shown a grid either at the top or at the bottom of the screen with a black dot in one of the boxes. Participants did not receive any information beforehand on what grid (upper or lower) was going to be shown to them. Participants then had to decide whether the dot in the grid was in the correct place (yes = “m” key/no = “x” key) based on the information from the arrows that had been presented. After two practice trials, participants performed 32 test trials. The number of errors was used as the dependent measure in the analyses.

In the Letter memory task (Morris and Jones, 1990), the third task used as a measure of updating ability, a number of letters was shown serially at the center of the screen. Each letter was shown for 2000 ms and participants did not know beforehand the number of letters that was going to be displayed. After two practice trials, 12 test trials were performed, in which the task was to recall the four letters most recently presented, in the correct order. After each trial, participants wrote down the letters they could recall in a test protocol. The number of correct letters in the correct numerical order was used for scoring of results. For easier interpretation in relation to the other executive tasks, the number of errors were used as dependent measure in the analyses.

#### Covariates

Age, sex (female = 0, male = 1), and years of education were used as covariates in the analyses.

### Statistical Analyses

**FIGURE 1:**
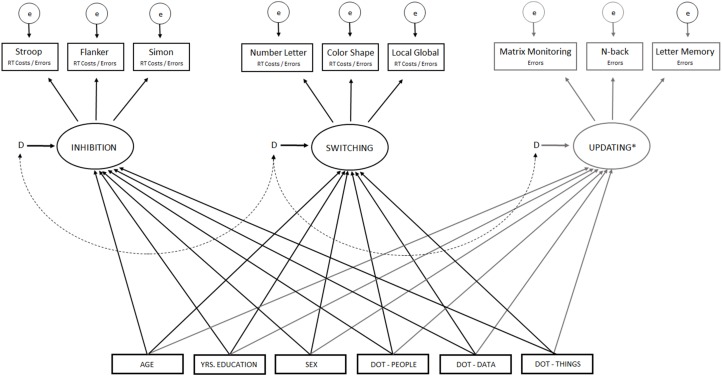
Illustration of the structural equation model(s) used in the present study. Covariance paths (not shown) were drawn between each predictor variable. Predictors and error terms (D) for latent variables were correlated by double headed arrows. ^*^Updating as dependent variable was only included in analyses of error rates since processing cost in RT is not calculated for these tasks. Occupational complexity working with data, people, and things was estimated according to Dictionary of Occupational Titles (DOT).

In the calculation of processing costs in the inhibition and switching tasks, only RTs from correct responses were used. Outliers were excluded according to the 3 Interquartile range rule (3 IQRs). Structural equation modeling (SEM) was used to analyze associations between constructs using IBM SPSS AMOS 24 (Arbuckle, 2016) with full information maximum likelihood (FIML) estimation. For model/s used, see Figure 1.

In a first model, we investigated the effects of the DOT variables on processing cost in RTs by including paths from each of the DOT variables to the latent construct of inhibition and switching. Processing costs in RTs for the Stroop task, Flanker task, and Simon task were used as indicators for the latent variable inhibition, and processing costs in RTs for the Number-letter task, the Local-Global task, and the Color-Shape task, were used as indicators of switching ability. For the updating tasks, processing costs in RTs are not calculated, and thus this updating was not included in the first model. Age-, sex-, and educational- influences were controlled for in the model by including paths from age, sex and education to each of the latent constructs. In a second model, we investigated the effects of each of the DOT variables on error rates in each executive construct (inhibition, switching, updating). Errors rates in the N-back task, the Letter-Memory task, and Matrix monitoring, were used as indicators of updating ability. The same predictors of performance were used as in the first model. In both models, predictors, as well as the latent constructs, were correlated by double headed arrows. We used the χ^2^*/df* value as the indicator of model fit supplemented by fit indices proposed by Hu and Bentler (1999) that a Root Means Squared Error of Approximation (RMSEA) and Comparative Fit Index (CFI) reveal important information about model fit.

## Results

Sample characteristics for predictor variables used in structural equation models can be seen in Table 2.

**TABLE 2:** Characteristics of participants used in the present study.

**Sample Charateristics**	**Mean**	***SD***	**Skewness^a^**	**Kurtosis^b^**
Age	65.6	5.8	–0.69	0.16
Years of Education	13.5	4.5	–0.03	0.28
DOT – Data	3.5	1.7	–1.02	0.10
DOT – People	2.9	2.3	0.67	–0.90
DOT – Things	2.4	2.6	0.30	–1.63
Females (%)	60.4			

*DOT = Dictionary of Occupational Titles. DOT values have been coded so that a higher value reflects higher occupational complexity. ^*a*^Std. Error 0.162–0.165, ^*b*^Std. Error 0.323–0.328.*

As can be seen, skewness for all predictor variables were below 2 and for kurtosis it was below 7, which are suggested thresholds in the literature (Finney and DiStefano, 2006). Sample mean for years of main occupation was 24.8 (*SD* = 10.8). Descriptive data of processing cost in RTs and error rates for each executive task, is presented in Table 3. As shown, values of skewness and kurtosis indicates normally distributed data for all executive scores.

**TABLE 3:** Performance on the executive tasks.

**Variable**	**Mean**	***SD***	**Skewness^a^**	**Kurtosis^b^**
***Processing Cost (RTs in ms)***				
Stroop	260.2	186.8	0.62	0.78
Flanker	101.0	55.2	0.51	–0.09
Simon	38.8	36.7	0.01	–0.09
Number Letter	864.8	662.7	0.80	1.01
Color Shape	170.9	137.8	0.63	0.01
Local Global	252.0	317.4	0.57	0.65
***Error Rates (raw scores)***				
Stroop	5.5	5.4	1.45	1.66
Flanker	1.8	1.8	1.18	1.19
Simon	4.8	4.3	1.36	1.42
Number Letter	3.5	3.4	1.31	1.37
Color Shape	5.5	3.8	1.24	0.92
Local Global	4.6	4.8	1.69	2.78
Matrix Monitoring	9.9	4.1	0.42	–0.26
N-back	8.0	5.1	0.75	0.70
Letter Memory	16.2	7.2	0.28	–0.34

*^*a*^Std. Error 0.167–0.181, ^*b*^Std. Error 0.333–0.360.*

Next, results from SEM analyses of the first model, with processing costs in RTs as dependent measures, indicated reasonable fit (CFI = 0.94, RMSEA = 0.03, χ^2^*/df* = 1.26). For CFI, values equal or greater than 0.95 indicates acceptable model fit, and for RMSEA a value of 0.06 or less is equal to good model fit (Browne and Cudeck, 1993; Hu and Bentler, 1999). For normed Chi-square values, suggested upper thresholds differ between 2.0 (Tabachnick and Fidell, 2007) and 5.0 (Wheaton et al., 1977). Thus, based on these suggested thresholds, model fit values were indicative of a good model fit with regard to RMSEA and χ^2^/*df*, and almost acceptable with respect to CFI. Both unstandardized and standardized regression weights as well as standard errors together with *p*-values for the predictor variables included in model are provided in Table 4.

**TABLE 4:** Regression weights of predictors in the model that included age, years of education, sex, and the DOT variables as predictors of processing costs in the executive tasks.

		**Processing**				
		**Cost**	**β**	***B***	**S.E.**	***P***
Age	→	Inhibition	0.517	10.167	2.204	**<0.001**
Age	→	Switching	0.151	6.677	4.974	0.179
Years of Education	→	Inhibition	–0.025	–0.631	2.839	0.824
Years of Education	→	Switching	–0.020	–1.129	6.380	0.860
Sex	→	Inhibition	–0.085	–19.49	24.602	0.428
Sex	→	Switching	–0.097	–50.435	56.611	0.373
DOT – Data	→	Inhibition	–0.107	–7.153	8.424	0.396
DOT – Data	→	Switching	0.211	31.754	20.498	0.121
DOT – People	→	Inhibition	–0.018	–0.877	6.188	0.887
DOT – People	→	Switching	–0.069	–7.494	14.028	0.593
DOT – Things	→	Inhibition	–0.148	–6.535	4.814	0.175
DOT – Things	→	Switching	0.011	1.115	10.769	0.918
Stroop	←	Inhibition	0.599	1		
Flanker	←	Inhibition	0.203	0.099	0.050	**0.047**
Simon	←	Inhibition	0.309	0.100	0.034	**0.003**
Number Letter	←	Switching	0.384	1		
Color Shape	←	Switching	0.446	0.242	0.089	**0.007**
Local Global	←	Switching	0.505	0.632	0.228	**0.006**

*β = Standardized regression weight. *B* = Unstandardized regression weight. S.E. = Standardized error of *B.* Sex was coded as female = 0 and male = 1. Bold values denote statistical significance at the *p* < 0.05 level.*

As can be seen, none of the occupational complexity variables could significantly predict processing costs for inhibitory control and switching ability in the first model. Only one predictor, higher age, was related to higher processing costs on inhibition.

Next, we tested the second model that included error rates for inhibition, switching, and updating as dependent variables. Model fits for this model were: CFI = 0.98, RMSEA = 0.02, χ^2^*/df* = 1.11. Thus, the model was good with regard to all fit indices. Regression weights, standard errors, and *p*-values for the predictor variables are provided in Table 5.

**TABLE 5:** Regression weights of predictors in the model that included age, years of education, sex, and the DOT variables as predictors of errors in the executive tasks.

		**Errors**	**β**	***B***	**S.E.**	***P***
Age	→	Inhibition	0.579	0.225	0.052	**<0.001**
Age	→	Switching	0.320	0.095	0.029	**<0.001**
Age	→	Updating	0.410	0.184	0.039	**<0.001**
Years of Education	→	Inhibition	0.092	0.046	0.045	0.312
Years of Education	→	Switching	–0.175	–0.067	0.036	0.062
Years of Education	→	Updating	–0.075	–0.043	0.050	0.382
Sex	→	Inhibition	–0.149	–0.681	0.405	0.093
Sex	→	Switching	–0.118	–0.412	0.306	0.179
Sex	→	Updating	–0.113	–0.595	0.431	0.168
DOT – Data	→	Inhibition	–0.062	–0.081	0.133	0.541
DOT – Data	→	Switching	–0.106	–0.107	0.104	0.305
DOT – Data	→	Updating	–0.210	–0.320	0.149	**0.031**
DOT – People	→	Inhibition	–0.100	–0.095	0.099	0.335
DOT – People	→	Switching	–0.248	–0.181	0.080	**0.023**
DOT – People	→	Updating	–0.188	–0.207	0.109	0.058
DOT – Things	→	Inhibition	–0.070	–0.061	0.076	0.421
DOT – Things	→	Switching	0.002	0.001	0.059	0.980
DOT – Things	→	Updating	–0.027	–0.028	0.084	0.742
Stroop	←	Inhibition	0.415	1		
Flanker	←	Inhibition	0.597	0.497	0.117	**<0.001**
Simon	←	Inhibition	0.597	1.161	0.269	**<0.001**
Number Letter	←	Switching	0.498	1		
Color Shape	←	Switching	0.647	1.485	0.290	**<0.001**
Local Global	←	Switching	0.507	1.424	0.306	**<0.001**
Matrix Monitoring	←	Updating	0.636	1		
N-back	←	Updating	0.568	1.114	0.186	**<0.001**
Letter Memory	←	Updating	0.494	1.384	0.253	**<0.001**

*β = Standardized regression weight. *B* = Unstandardized regression weight. S.E. = Standardized error of *B.* Bold values denote statistical significance at the *p* < 0.05 level.*

Results from the second model (with error rates) revealed a relationship between complexity with data (DOT–data) and updating (standardized β = −0.210, S.E. = 0.149, *p* = 0.031), and between complexity with people (DOT–people) and switching (standardized β = −0.248, S.E. = 0.080, *p* = 0.023). With regard to paths drawn between occupational complexity factors, there was a significant correlation between DOT–data and DOT–people (*r* = 0.52, *p* < 0.001) and between DOT–things and DOT–people (*r* = −0.21, *p* = 0.002). DOT–data and DOT–things were uncorrelated (*r* = −0.01, *p* = 81). More years of education was, as expected, positively correlated to complexity working with data (*r* = 0.26, *p* < 0.001) and people (*r* = 0.25, *p* < 0.001), whereas it was negatively correlated to complexity working with things (*r* = −0.15, *p* = 0.029). As can be seen in Tables 4, 5, in both models all executive tasks loaded significantly on the latent variable representative for their respective construct.

### Additional Analyses

Although both executive functions and fluid reasoning are mediated by frontal lobe functioning, and both are considered to be core aspects of intelligence (Decker et al., 2007), we also examined if relationships would persist after including a measure if fluid intelligence (*Gf*) as predictor into the model. Results from the 12-item short form of the non-verbal Raven Advanced Progressive Matrices Test (Arthur and Day, 1994) were used as a proxy *Gf*. This short form has essentially the same measuring properties as the original form (*r* = 0.90). Zero-order correlations between the cognitive variables showed that higher *Gf* correlated significantly with lower error rates in all tasks (*r* ranging from −0.23 to −0.52, *p* < 0.01).

Model fits were good: CFI = 0.98, RMSEA = 0.02, χ*2/df* = 1.13. After adjustment of *Gf*, the association between higher complexity of working with people (DOT–People) and lower switching errors remained (standardized β = −0.208, S.E. = 0.08, *p* = 0.047) whereas the relationship between complexity with data (DOT–data) and updating was no longer significant (standardized β = −0.130, S.E. = 0.14, *p* = 0.13).

## Discussion

Although previous studies have demonstrated a relationship between professions with a higher level of occupational complexity and cognitive functioning (e.g., Fisher et al., 2014; Vemuri et al., 2014; Pool et al., 2016), only a few have included tests that tap executive functioning (see Adam et al., 2013; Feldberg et al., 2016), and no study has, to our knowledge, used a broader set of executive tasks aimed to measure all three executive components. The need of research that investigates possible effects of occupational complexity on executive processes is not only important since functional attentional resources are needed in everyday life, but also important as aging is often accompanied with a decline in executive functioning (e.g., Connelly et al., 1991; Salthouse and Miles, 2002; Zelazo et al., 2004; Treitz et al., 2007). Thus, the aim of this current study was to investigate if occupational complexity in main lifetime occupation, according to DOT classifications, was related to performance in executive functioning (switching, inhibition, updating). Data emanated from a study sample (“Successful aging–A study of how bilingualism and choice of occupation contribute to preserve attention and memory across the adult life span”) that was between 50–75 years at time of measurement. Results from structural equation models revealed a significant relationship between occupational complexity working with data and performance (less errors) in the updating component of executive functioning. Complexity working with people was related to performance (less errors) in task-switching. Additional analyses revealed that the relationship between complexity with people and lower switching errors persisted also after the inclusion of *Gf* in the model. However, even if results are promising with regard to the possibility that executive functions can be trained, due to the design of the present study we cannot draw any conclusions about causality. Cognitive abilities may also act as predictor of occupational choice. Even if *Gf*, known to be extremely stable over the life course (see e.g., Rönnlund et al., 2015), was included as proxy of initial cognitive ability in some analyses, it does not adjust for the directionality between factors. Thus, results from this study must be interpreted from two alternative explanations.

The present demonstration that occupations with higher level of complexity with both people and data are associated to performance on aspects of attentional control, builds on results from previous “DOT-studies” that have suggested that higher levels of occupational complexity working with people (e.g., Andel et al., 2007; Smart et al., 2014) and with data (Correa Ribeiro et al., 2013; Smart et al., 2014) can be related to improved performance on global measures of cognitive functioning (e.g., MMSE). We did not find any relations to complexity working with things, which could indicate that this factor is not specifically related to any executive abilities. Similarly to us, Feldberg et al. (2016) found in their study on patients with MCI, that a lifetime occupation with high complexity working with data was related to better performance on two measures supposed to tap executive functioning: Analogies and Trail making test, and complexity working with people to better performance in Analogies. Working with things, however, was associated with performance in “Cubes,” a task that rather reflects visuospatial ability.

With regard to occupational complexity working with people, it should be stressed that significant results were found on accuracy scores in task-switching ability, but not for performance in updating or inhibition. An interpretation of this would be that task-switching ability is a necessary component and/or regularly trained when working in environments that include high complexity working with people. It is plausible that in occupations with high complexity working with people, you are often forced to shift/dual-task between factors such as being instructive, informative, communicative, pedagogical, listening, attentive, present, empathic, etc. In addition to this, when working with people you also need the capacity to be able to recognize perspectives and desires of others (see e.g., Ybarra et al., 2008) which put high demands on the cognitive system. Switching between these above-mentioned factors/tasks, which all in themselves can be very cognitively challenging, may therefore require a well-functioning switching ability. Switching ability may thus be one crucial factor for occupations with high complexity working with people as compared to complexity working with data and things, in general. It should be stressed that the association between complexity working with people and task-switching ability also persisted after adjustment of *Gf*. This is noteworthy since executive functioning is strongly related to intelligence (Decker et al., 2007). Thus, the association found seems to hold over and above *Gf*, at least from a psychometric perspective.

That greater complexity of work with data was related to better performance (less errors) in updating is a very intriguing result as well. As it seems, occupations that contain synthesizing, for instance, the factor defined to include the highest level of complexity working with data, is to a great extent related to updating ability. According to DOT classification (see https://occupationalinfo.org/appendxb_1.html), synthesizing reflects: “integrating analyses of data to discover facts and/or develop knowledge concepts or interpretations.” It does seem reasonable that occupations that involve analytical thinking, and to be able integrate data for knowledge development, can be related to the ability to constantly monitor and evaluate incoming information for task-relevance and revise information in working memory based on this, which is an important aspect of “updating ability” (Morris and Jones, 1990; Miyake et al., 2000). Similarly, the second highest rating in DOT on complexity working with data reflects “coordinating” and is claimed to include: “determining time, place, and sequence of operations or action to be taken on the basis of analysis of data; executing determinations and/or reporting on events.” Certainly occupations that include such demands put demands on the updating function. That results showed no relationship between complexity working with things and any executive component, which may just reflect that such occupations are not specifically related to the executive components included in this study.

In this study, we found that many participants performed with high accuracy in several tests, as indicated by low error rates on the mean level. This was especially evident in the inhibition and switching tests. This finding may indicate a ceiling effect, and that there is a risk of too little variance in the outcome measures to detect differences between participants. However, with possible exception of the flanker task, standard deviations of the tests showed that there was sufficient variance around the mean to justify the inclusion of the test results into the analyses. Skewness and kurtosis were also acceptable for all tests. However, it must still be stressed that many of the participants performed at a high level on many of the tests, and that many probably experienced some of the tests to be relatively easy to execute. Although we decided in advance to use only tests that are validated and commonly used in the literature, we cannot rule out the possibility that the result would have differed if we had used other tests of inhibitory control and task-switching than those in the present study. It may therefore be of interest for future studies to include other measures of executive function with even higher sensitivity and greater degree of difficulty than those used in the present investigation.

Results from this study provide new and important knowledge to this line of research. The present demonstration of associations between dimensions of occupational complexity and aspects of executive functioning, builds on previous results and *may* suggest that occupational complexity is a factor that also may promote our attentional resources. So far, many cognitive training interventions have failed to find far transfer effects (see summary in Sandberg, 2014). That is, better performance in executive tasks other than those closely related to what has been trained. However, in this study we may have come closer to identifying factors that can generate far transfer effects to executive functioning by demonstrating associations between “real-life cognitive training” and performance in the lab. Such finding could have important implications both from an individual and a societal perspective. First, to be able to boost our attentional resources may not only make us better suited to cope with everyday mental demands, it may also reduce stress levels and plausibly also increase self-confidence in many situations. Second, it is widely recognized that aging is accompanied by a decline in several cognitive functions (Bäckman et al., 2005) including executive functioning (see e.g., Salthouse and Miles, 2002; Zelazo et al., 2004; Treitz et al., 2007). Although results from this study are based on correlational data, and do not reveal any information on cognitive change, results can be indicative of factors that can be beneficial for executive processing, both in the short and long term. Thus, the next step is to investigate longitudinal effects of occupational complexity on the executive processes, information still missing in the literature.

In this study, we used both “RT costs” and errors rates as measures of switching ability and inhibitory control. It should be noted though that RTs are more often used in the literature for such tasks. However, for task-switching, we only found effects on error rates and not on processing cost in RTs (for complexity working with people). As participants were instructed not only to respond as fast as possible, but also to respond as accurately as possible, it is plausible that high priority was given for accuracy (as indicated by high proportion correct responses overall). Thus, participants with a history of high level of complexity working with people seem to be better suited to minimize errors rates in switching between tasks, although still able to do so without showing increased processing costs in RTs as compared to participants with lower level of complexity working with people, finding that just as well may be indicative of higher expertise.

Some studies argue that executive functions are mostly driven by genetics and thus a very heritable trait (Friedman et al., 2008). Even though we found relationships that may suggest that some aspects of executive functioning can be influenced by environmental stimulation, we cannot, as previously noted, exclude the possibility that variations in cognitive ability make individuals more prone to select some occupations (see e.g., Theorell et al., 2019). Although environmental factors may have a greater impact on cognitive functioning than the reverse (Kohn and Schooler, 1973), and that there is support for the notion that intellectual engagement earlier in life plays a positive role for cognition, as suggested in the concept of cognitive reserve (Stern, 2002) and the environmental complexity hypothesis (Schooler, 1984), we are still limited to cross-sectional data in this study. Future studies with longitudinal analyses could be useful also in this regard.

We used several established neuropsychological tests in this study. However, future studies examining the extent to which occupational complexity may influence executive functions should consider including measures with high ecological validity as well. Tests that may expand upon naturalistic environment and situations may generate more knowledge to the field to predict everyday executive functioning. Finally, it should be noted that occupational complexity is a hypothetical framework, and that the DOT-codes used as indicators of complexity levels are general values. Although the occupational title may be the same, most often every work has several unique characteristics that in turn may put different demands on cognitive abilities. Thus, our results must be interpreted with this in mind. In addition, DOT-codes are derived from US census data, and for some occupations it may not be a straightforward procedure to use these in a Swedish context. Although it has been confirmed that DOT-codes can be used as a valid measure of occupational complexity (Andel et al., 2005; Potter et al., 2008), differences should be considered when interpreting our findings.

## Concluding Remarks and Further Research

In sum, this study demonstrates that occupational complexity factors can be related to performance in executive control tasks. By using DOT classifications, three dimensions of occupational complexity were included in the analyses: complexity working with data, complexity working with things, and complexity working with people. Higher complexity of working with data was related to lower error rates in the updating component of the executive control system, and higher rates in complexity working with people with less errors on the task-switching component. Working with things was not related to performance in any of the executive dimensions included in the analyses. Although results from this study may have important implications both from an individual and societal perspective, further research is needed to replicate these findings. Future studies would also benefit from a longitudinal assessment to investigate the directionality between occupational complexity and executive functioning, and consider the use of tests with high ecological validity to be able investigate whether benefits can be translated to everyday executive functioning.

## Ethics Statement

The study has been approved by the Regional Ethics Committee at Umeå University (2016/101-31Ö). All subjects gave written informed consent in accordance with the Declaration of Helsinki.

## Author Contributions

DS, IP, JL, and PH developed the research questions and wrote the sections “Introduction,” “Materials and Methods,” “Results,” and the “Concluding Remarks and Further Research.” DS performed the formal analyses. All authors have contributed equally.

## Conflict of Interest Statement

The authors declare that the research was conducted in the absence of any commercial or financial relationships that could be construed as a potential conflict of interest.
